# Acute Parenchymatous Degeneration of the Kidneys

**Published:** 1883-04

**Authors:** William Henry Porter

**Affiliations:** Associate Professor of Normal and Pathological Histology at the New York Post Graduate Medical School, Professor of Surgical Pathology and General Surgery at the Columbia Veterinary College, Curator of the Presbyterian Hospital Museum, Etc., Etc.


// THE JOURNAL

OF

COMPARATIVE MEDICINE AND SURGERY.

VOL. IV. APRIL, 1883. No. 2.

### ORIGINAL COMMUNICATIONS.

### ART. VIII.—ACUTE PARENCHYMATOUS DEGENERATION OF THE KIDNEYS.

BY WILLIAM HENRY PORTER, M.D.,

*Associate Professor of Normal and Pathological Histology at the New York Post Graduate Medical School, Professor of Surgical Pathology and General Surgery at the Columbia Veterinary College, Curator of the Presbyterian Hospital Museum, Etc., Etc.*

A PARENCHYMATOUS degeneration of the kidney is a morbid condition which every veterinarian should be familiar with, and be able to recognize in its early stage.

In this brief article it will be impossible to consider the subject in full, but the object is rather to make the leading points of this disease prominent so that an early and acurate diagnosis can be made.

First, what are the leading features of the lesion? It is characterized by a marked alteration in the epithelial elements of the renal glands, but no appreciable change in their blood vessel or interstileal tissue.

*Etiology.*—This form of change is met with in human medicine in connection with, or as a complication of, several diseases, which are accompanied by a long continued elevation of body temperature, namely, pneumonia, typhus, typhoid and yellow fever, puerperal fever, scarlet fever and diptheria, bad cases of cholera, acute yellow atrophy of the liver, and is also the result of arsenic and phosphorous poisoning, and some cases are said to develop without any appreciable cause.

By carefully considering this list of diseases you will readily see that it comprises those which must have had some original poison as the exciting cause, and one which of necessity would interfere greatly with the normal physiological tissue metamorphosis; consequently we have in the blood to be eliminated both the original poison and varying quantities of the products of incomplete tissue transformation. I believe these incomplete products are either the result of diminished or increased physiological activity, or both, probably, more correctly speaking, diminished in reference to perfection, but increased both in rapidity and quantity. The result of such a change necessarily produces great irritation to the whole system, and by the combined action of the original poison and the effete material circulating in the blood, we have produced the phenomena known as elevation of temperature.

Having this undue amount of deleterious material in the system, and the kidneys together being one of the great eliminating organs, they make an attempt to free the system of these irritating materials and the result is,that the renal epithelial protoplasm becomes unduly irritated, swollen, and finally filled with minute particles of these irritating substances, which ultimately destroys the vital properties of the cell protoplasm, and if continued long enough will cause them to undergo a fatty transformation. As the disease advances the epithelium steadily degenerates, and finally may loose its hold upon the basement substance and is swept off by the current of urine from above and appearing in the urine is known as cast matter or casts. The result of this change will be that we have the tubes in the kidney not lined by epithelium, but having for their walls the remaining basement membrane. I am inclined to believe that this form of disease is not a truly inflammatory process, but one due to the primary and continued irritation of the above mentioned excretory products. Just what the chemical nature of the products may be is a matter yet to be settled.

At this point the question may rise what are casts? There are two principal types, the blood and hyaline, and in connection with the latter there is a large modification as may be seen by a single glance at the following table :

| | | | |
|---|---|---|---|
| CASTS. | BLOODY. | | |
| | HYALINE TYPE. | *Small.* | Hyaline. |
| | | | Epithelial. |
| | | | Nucleated. |
| | | | Finely granular. |
| | | | Coarsely granular. |
| | | | Fatty. |
| | | | Tubular. |
| | | | Cork screw. |
| | | *Large.* | Hyaline. |
| | | | Epithelial. |
| | | | Nucleated. |
| | | | Finely grnnular. |
| | | | Coarsely granular. |
| | | | Fatty. |
| | | | Tubular. |
| | | | Cork screw. |

The blood cast is simple, and easily understood. It is produced by an exudation of all the constituents of the blood, and a matting together and an entanglement of the blood corpuscles by fibrin elements in the lumen of the uriniferous tubules, so that they are discharged from the tubes in masses representing perfect casts of the same, and are found in the urine as little plugs of blood corpuscles with parallel sides and rounded ends.

The hyaline cast is not so well understood, but it is generally accepted that a peculiar gelatinous substance is thrown out into the uriniferous tubule, and if discharged from the same independent the epithelium is known as a hyaline cast, but with attached epithelium in various stages of retrograde change, produces the various forms above tabulated.

By an epithelial cast is meant one in which the epithelium has adhered to this hyaline plug and been separated from the basement membrane while still retaining the appearance of epithelium.

The nucleated is where the protoplasm has been obliterated, and only the nuclei can be recognized adhering to the hyaline substance.

The fine granular is where the protoplasm and nuclei are obscured or replaced by fine granular particles.

The coarse granular is where protoplasm and nuclei are obscured or replaced by coarse granular particles.

The fatty is where the epithelial element are replaced by large or small fat droplets.

The tubular is a rare variety, and is formed by a plug of hyaline matter in the lumen and a thin ensheathining layer of the same behind the epithelium, and in this manner the epithelium is separted and discharged, and presents itself under the micro scope as a perfect ring of epithelial elements. The size of the cast will vary with the original size of the tube, and the amount of degeneration and dilitation of the same. The distinguishing characters of casts are their parallel sides and rounded ends.

In rare and exceptional cases there is twisting of the body of the cast, so that it resembles the spiral of a corkscrew. Prof. T. E. Satterthwaite first called my attention to this peculiar variety, and I will say at this point that we have sections of kidneys that show a peculiar spiral arrangement of the tubes, which easily accounts for this strange form of casts.

The waxy cast of some writers is not included in this table, as its existence is extremely doubtful and probably never occurs.

By the size of the casts we are, in a measure, enabled to determine the stage of the disease. If they are small it is the early part, if they are large it represents a later period. The early stage may be subdivided into two. In the first there will be small hyaline, epithelial and nucleated; in the second fine and coarsely granular and possibly fatty casts, all of the small type. Large hyaline, epithelial and nucleated casts indicate great primary severity. Large granular and fatty represent an advanced state of the lesion.

*Pathological Changes.*—If one of these kidneys be examined in the early stage it will be about normal in size, the capsule normal in thickness, non-adherent to the underlying renal tissue, which is left perfectly smooth after its enucleation from the capsule. The cut surface will present no marked appearances by which this lesion can be readily recognized by the naked eye. But when examined under the microscope the following changes will be found. The epithelium will present a cloudy, mucoid or freely granular appearance. The cells will be swollen and the lumen of the tubules occluded, and in the early stage, where there is rapid swelling of the epithelium with but little or no expansion of the capsule, the tubules will be tortuous. Later in the change the cut surface will appear paler than usual and the pyramids less distinct, and now the kidney will probably be larger and softer than normal. Under the microscope the granular degeneration of the cells is very well marked. Desquammating masses of cells, precisely like the casts found in the urine, are present in the tubes, and some of the tubes have completely lost their epithelial lining. This rapid disquammation and stripping of the tubes is especially marked in this form of disease occurring in the horse. There is usually greater softening of the horse's kidney than of the human, so that in some cases it becomes almost gelatinous. In other cases there is marked congestion of the kidney and a deposition of blood pigment in the intertubular spaces, and in some of the cases the kidney will remain quite firm.

*Symptoms.*—These are somewhat different in man and animals, in man occurring as they do in connection with severe disease, or as the result of some metalic poison, the symptoms are not often well marked at first. There may be a little œdema of the cellular tissue just under the inferior eyelids, or of the feet, which will first attract attention. This statement is especially true in arsenical poisoning of the chronic variety. But in connection with the severe diseases the scanty and high colored urine will lead to a critical examination of the same, and the finding of albumen and cast, common to this variety of lesion, will of necessity establish the diagnosis of paranchymatous degeneration of the kidneys, and indicates great intensity of the original disease.

But in equine practice this form of disease seems most frequently to occur after a period of rest, followed by active exercise, and as a result the sudden development of a weakness or partial paralysis of the posterior extremities, often increasing to a degree of total disability. At this time the animal gives evidence of a moderate chill, and falls into a profuse perspiration. As the disease advances the urine becomes more scanty. A strong urinous odor may be detected emanating from the animal, convulsions set in, which rapidly pass on to coma and death.

If the urine is carefully examined in such cases it will be found to contain large quantities of albumen and casts of the varying kinds above described, namely epithelial, nucleated, fine or coarsely granular, more frequently, however, the two latter, and larger masses of granular debris. The urine of the horse will have an alkaline reaction and a specific gravity above normal, perhaps as high as 1.030.

To test for albumen in the urine it must first be filtered, to remove the large amount of thick, tenacious mucus. This having been accomplished the filtrate is boiled, and a little acetic acid added to render the urine acid, as albumen is not precipitated in alkaline solutions; if a precipitate is produced which is not redissolved upon the application of a moderate quantity of nitric acid, but becomes more marked, then albumen is present. This, perhaps, is the most ready and practical test, and if pains are taken to look through the specimen in the test tube against a black background, as the sleeve of a coat, or a hat, or book, it proves a delicate and sure test. There are numerous other tests by which this can be confirmed, and as they are more troublesome and not so practical it seems hardly necessary to mention them in detail, but they will be found in the case department in connection with the case reported by Dr. Walton.

The casts, of course, can only be determined by the aid of the microscope.

With such rational and urinary symptoms as these the diagnosis becomes both easy and positive.

It is hard, however, to explain the accompanying paraplegia, and at one time it seemed quite rational to look upon the paralysis as due to a lesion of the cord which preceded that of the kidney. But the more the symptoms and lesions of the kidney are studied the more reasonable it seems to place the kidney lesion first and the paralysis as secondary, but why paraplegia should occur is at present hard to understand.

The cause of the renal degeneration seems to be due to the attempt on the part of nature to free the system of effete material, which probably is produced by the sudden activity after a period of rest. It may be that this causes the renal trouble, and the unexpected strain of active exercise after a period of rest affects the cord at about the same time, and that the two develop simultaneously, but are due to independent causes, and this concomitant paralysis may be a factor in making the renal lesion so grave.

These points which have been raised are those which every Veterinarian should investigate closely and record and publish until something more positive can be elucidated.

The term Azoturea is one that should be discarded as meaningless, for it has not been explained what is meant by it. To be sure the definition "nitrogenous urine," and "hyper-nitrogenous blood" has been applied to it, but fails to say whether it means that pure nitrogen gas is in the blood and urine, a rather absurd supposition, or an increased amount of albumen or of urea, and until it is positively stated which is meant, and some quantitative analysis appended to settle these points in question, it must of necessity remain in its present meaningless position.

*Treatment.*—This, of course, is to be based upon one grand principle, and that is to relieve the kidneys as much as possible, and also rid the system rapidly and easily of all effete and irritating substances. This can be accomplished in a measure by free purgation and diaphoretics, among which the sulphate of pilocarpine hypodermically injected stands first among the list of diaphoretics and eleterium first among the cathartics. The kidneys should not be neglected, but be kept at work by non-irritating diuretics, so that what epithelium remains sound may aid in freeing the system and yet be as little irritated as possible, and also to keep the degenerating epithelial elements from blocking the tubules, and thus damming back the urine. This can be accomplished by large draughts of water, digitalis, with heat and stimulation to the loins. In this way a larger quantity of water passes through the kidneys and thus diminishes the density of the fluid, and relieves as much as possible the irritation of the effete material. Nitrate of potash should never be given in such cases, as it acts in directly the opposite direction, and is simply adding fuel to the fire, and will almost inevitably cause death. It seems quite reasonable to consider the constant and free use of this salt in veterinary practice as one grand cause in weakening the kidneys and rendering these renal lesions so common. Tonics and the most easily digested foods should not be forgotton.

From close observation one can easily satisfy himself that as a general rule what is deleterious to man is very apt to be in the animal kingdom, even though there are some exceptions.

